# Supplementary material for: Are carbon emissions trading and green financial instruments synergistic? -Comprehensive quantitative research based on content analysis
Source: PLoS One. 2024 Mar 7;19(3):e0298601. doi: 10.1371/journal.pone.0298601 (PMC10919601; doi:10.1371/journal.pone.0298601)
Supplement: S1 Data — (ZIP) [file pone.0298601.s001.zip › Data/地级市Z_F工作报告.zip!/║■▒▒/╨ó╕╨╩╨/└┤╫╘╣1⁄2╓┌║┼í╛┬φ┐╦╩2╛▌═°í┐_2013.docx]

各位代表：

　　现在，我代表市人民政府向大会报告工作，请予审议，并请市政协委员和列席人员提出意见。

　　一、2012年工作回顾

　　刚刚过去的2012年，是全市经济社会发展进程中极不平凡、极不寻常的一年。在省委省政府和市委正确领导下，市政府团结带领全市人民，承前启后、继往开来，抢抓机遇、攻坚克难，团结一致、拼搏奋进，成功经受住了错综复杂外部环境、宏观经济下行压力等多重考验，继续保持了经济持续较快增长、社会事业全面进步、人民生活水平不断提升、社会和谐稳定的良好态势，圆满完成了市五届人大一次会议确定的目标任务。

　　——综合实力实现新跨越。经济总量迈上千亿元大关。预计全年实现生产总值1100亿元，比上年增长11.5％；全社会固定资产投资950亿元，增长30%；社会消费品零售总额526亿元，增长15.6%；财政总收入102.2亿元，增长31.9%；地方公共财政预算收入69.5亿元，增长34.6%。工业经济做强做大。规模以上工业企业达到950家，新增113家；实现增加值480亿元，增长17.5%。新增销售过亿元企业115家、过10亿元企业7家。新增国家驰名商标3件。新增高新技术企业26家，高新技术产业实现增加值115亿元，增长22%。建筑业实现增加值52亿元，增长17.2%。农村经济稳步发展。粮食产量实现“九连增”。160家市级以上农业产业化重点龙头企业实现产值（经销额）290亿元,增长17%。农机化率上升到72.1%。完成各类水利工程投资20.2亿元，除险加固水库66座，“三万”活动扩挖塘堰3万口，新改建县乡村公路519公里，新解决农村26万人饮水安全问题，实现3.85万贫困人口基本脱贫。第三产业快速增长。新增限额以上商贸企业173家。一批重大物流项目开工建设。金融机构存、贷款余额分别达到1163.5亿元、552.5亿元，增长20.7%和18.2%。成功发行10亿元城投债券。旅游接待人数1200万人次、综合收入70.6亿元，分别增长20%和21.9%。房地产市场保持基本稳定，住房公积金扩覆1.12万人。电力、烟草、邮政通信、石油、盐业等行业实现新的发展。

　　——发展后劲得到新增强。以“项目建设年”为抓手，以改革创新为动力，加强市县乡村四级统筹，发展活力倍增。重大项目扎实推进。策划推进一大批重点项目，投资总额1820亿元。在建亿元以上项目301个，其中新开工亿元以上项目192个、完成投资242亿元，分别比上年增长1倍以上。争取国家、省项目资金10.6亿元。对外开放不断扩大。内联引资650亿元，增长85.6%。新引进投资过10亿元的项目32个，其中过50亿元的7个。新批外商投资企业18家，实际利用外资2.4亿美元，增长17.7%。新增出口企业56家，出口总额6.4亿美元，增长68.9%。市政府与汕头市政府、广州市天河区政府签订战略合作框架协议。改革创新取得突破。建立省级以上研发平台45家、院士工作站6家。圈域一体化〔1〕深入推进，新承接武汉产业转移项目625个，到位资金266.6亿元。市县两型社会示范区规划提升，建设提速。大别山试验区19个“点中点”示范项目〔2〕顺利推进。营业税改征增值税试点顺利启动，国有资产、文化体制、事业单位分类、集体林权制度等改革不断深化。市直“三区”建设加快。孝感高新区获批国家高新区，31个项目竣工投产，33个项目开工建设，税收过千万元企业达到8家。临空经济区总体规划提升，“两横一纵”骨干路网基本建成，申通物流项目开工建设。双峰山旅游公路开工。县市区竞相发展。孝南新增税收过亿元企业2家，汉川电厂三期第一台100万千瓦机组并网发电，应城富邦科技上市获中国证监会批准，云梦城市饮水工程等10个对接孝感项目快速推进，安陆拓展开发区面积10平方公里，大悟开工建设高铁经济新区，孝昌高标准规划建设“三河六岸”〔3〕县域经济蓬勃发展。

　　——城乡面貌呈现新变化。以“全域孝感”〔4〕理念统领新型城镇化、城乡一体化和新农村建设，城镇化率进一步提升。规划统筹力度加大。在全省率先编制市域统筹规划，“1531”城乡空间发展体系〔5〕全面形成。孝感城区建设提速。实施市政工程项目54个，完成投资35.3亿元。槐荫公园二期、人民广场二期等十大城建项目进展较快。东城新区行政中心主体工程完工，南城新区北京南路、澴川风景区等项目开工建设。一批改善市民居住环境的城建项目按期完工，新改建城区道路89公里，硬化背街小巷和破损路面4万平方米，新增绿地15万平方米。城镇建设整体推进。6个县市城区共实施城建项目127个，完成投资23.2亿元。汉川市民服务中心、应城刘杨新社区、云梦“三湖连河”〔6〕、安陆中心城区改造、大悟乐冲新社区、孝昌博士湾等一批城建工程加快推进。启动建设城市、城镇和农村三类新社区43个。京广高铁大悟段及孝感北站投入运营，大别山红色旅游公路、316国道外迁等重大交通项目开工建设。城乡综合管理成效明显。市县城区“四城同创”、“一线一点”〔7〕建设和文明卫生村镇创建工作扎实起步，城乡环境卫生、城市交通秩序等综合整治力度加大，市容市貌、乡村环境明显改观。生态环境治理加强。实施减排项目38个，关闭高污染、高能耗企业5家。在全省率先建立重点污染源监控平台，建成PM2.5〔8〕大气灰霾监测站。植树造林18万亩。万元生产总值综合能耗、二氧化碳和主要污染物排放量完成省定目标任务。

　　——民生福祉得到新改善。切实念好衣食住行、业教保医“八字经”，市政府承诺的十件实事全面完成。城镇居民人均可支配收入1.81万元、增长13.9%，农民人均纯收入8080元、增长15%。就业社保增覆扩面。新增城镇就业5.5万人、下岗再就业1.8万人、转移农村劳动力6.9万人。社会保险参保人数增加到473万人次。城乡居民社会养老保险参保率、城镇居民医保参保率、新农合参合率分别提高到95%、99%和98.8%，在全省率先推行新农合结算“一卡通”。新建4个县级社会福利中心。残疾人得到各类救助4万多人次。56.2万农村60岁以上老人享受基础养老金待遇，市区高龄老人补贴发放到位。新建各类保障性住房8753套，新增廉租住房租赁补贴3980户，改造棚户区1.15万户、农村危房1.09万户，恢复重建因灾倒房321户。社会事业全面进步。精神文明建设不断加强，成功进入全国未成年人思想道德建设先进市行列。加强人才引进和培养，招硕引博54人。新改扩建幼儿园50所，建成装备标准化合格学校423所。医改工作取得实效，孝昌、应城公立医院改革试点顺利推进。市中心医院新门诊大楼主体工程完工，7个县级医院中央投资项目竣工，免费救治晚期血吸虫病人448人次。成功举办市第四届运动会、第九届孝文化节、第五届省楚剧艺术节。软件正版化积极推进，电视栏目全面改版，对外宣传工作得到加强。计生家庭奖励政策实现全覆盖，出生人口性别比控制在省定责任目标之内。国防教育、民兵预备役、国家安全、人民防空工作有新进展，民族宗教、外事侨务、老龄、妇女、儿童、对台、地方志、档案、保密、机关事务管理工作取得新成绩。社会管理创新深入推进。63个社区完成“1+X”体系〔9〕规范化建设，市县主城区实现网格化管理全覆盖。压警情、控发案，社会治安秩序良好。解决信访问题1.8万件，化解信访积案243件。安全生产、食品药品、产品质量、市场物价监管进一步加强。

　　——行政效能实现新提升。按照省统一部署,认真开展“喜迎十八大、争创新业绩”主题实践活动，深入推进“解放思想大讨论”、“纠风正纪促跨越”，认真组织政风行风评议、电视问政、治庸问责等活动，部门服务意识增强。依法行政水平不断提高。法律援助、司法救助力度加大，“六五”普法〔10〕有效推进，法治孝感建设成效明显。国土资源、城乡规划、财政资金、国有资产转让等议事决策程序进一步规范。办理人大代表建议111件、政协委员提案306件，满意和基本满意率达100%。政府办事效率进一步加快。坚持开短会、发短文、讲短话、简办事，讲节俭。推动审批再清理、平台再创造、流程再创新，减少行政审批项目20项。市本级开通重大产业项目、基础设施项目审批“绿色通道”。廉政建设深入推进。源头治腐“十个全覆盖”〔11〕示范点工作取得新进展，全程网上招投标系统投入使用。政府投资项目全程跟踪审计制度不断完善，开展惠农政策落实、保障房建设、教育乱收费、医疗领域不正之风等15个专项治理。

　　各位代表，回顾过去的一年，我们感到，做好政府工作，必须坚持发展是第一要务，扩大总量，提升质量，推动全市经济社会科学发展、跨越式发展；必须坚持解放思想，“三情”结合，用改革创新的办法破解发展难题；必须坚持市域统筹，明确各地发展定位，充分调动干事创业激情，在应对挑战中奋发有为、争先进位；必须坚持以人为本，不断提高群众生活质量和幸福指数，让发展成果惠及全市人民；必须坚持勤政廉政，打造落实链条，以务实高效的作风推进政府工作。

　　奋斗历经艰辛，成绩来之不易。这是省委省政府和市委正确领导的结果，是市人大、市政协大力支持监督的结果，是全市人民团结奋斗的结果。在此，我代表市人民政府，向全市人民，向各位人大代表、政协委员，向离退休老同志，向各民主党派、工商联、无党派人士、人民团体和社会各界，向驻孝部队、武警官兵、政法干警，向所有在孝的投资者、劳动者、建设者和关心支持孝感发展的海内外朋友，表示崇高的敬意和衷心的感谢！

　　在看到成绩的同时，我们也清醒地认识到，政府工作与上级的要求和全市人民的期待还有差距：一是经济实力与武汉城市圈副中心城市的战略定位不完全相称，经济总量不大，支撑跨越发展的大项目、好项目不多。二是经济发展的结构性问题仍比较突出，工业化率和城镇化率低于全省平均水平，农业抵御风险的能力仍然脆弱，第三产业特别是服务业发展相对缓慢，市本级发展还不快。三是财政收入规模和质量有待提高，地方公共财政预算收入占生产总值的比重、税收占地方公共财政预算收入的比重均低于全省平均水平。四是城乡居民收入水平不高，部分群众生活困难，基本公共服务体系还不健全。五是政府系统解放思想的力度不够，推进各项改革率先突破的办法不多，担当意识、工作标准、落实力度都有待进一步提升。对此，我们将采取有效措施加以解决。

　　二、2013年工作安排

　　2013年是全面贯彻落实党的十八大精神的开局之年，是实施“十二五”规划承前启后的关键一年，是为全面建成小康社会奠定坚实基础的重要一年，也是孝感建市20周年。党的十八大提出到2020年实现全面建成小康社会的宏伟目标，省委省政府要求今后五年要在中部地区率先全面建成小康社会，孝感作为建设中的武汉城市圈副中心城市，必须进一步发挥优势，奋发进取。对照小康社会6大类23项具体指标分析，我市已有11项指标达到小康标准值，7项指标经过努力能够提前实现，但人均生产总值、第三产业占比、企业研发投入占生产总值的比重、单位生产总值能耗、环境质量指数5项指标距离目标值差距较大。当前，孝感正处在重要战略机遇期和黄金发展期，多重机遇叠加，多种优势并存，一批拉动经济增长的大项目相继开工和竣工投产，经济社会发展态势良好，全市上下谋发展、促跨越的强大气场已经形成，我们有信心、有能力、有条件发展得更好，发展得更快。

　　根据市委部署安排，2013年政府工作总的要求是：以党的十八大和省十次党代会精神为指导，高举中国特色社会主义伟大旗帜，深入贯彻落实科学发展观，围绕建设武汉城市圈副中心城市、打造“湖北的苏州”〔12〕和建设“魅力孝感”总体战略，大力推进“五个跨越”、加快建设“五个城市”〔13〕，坚持工业化、信息化、城镇化、农业现代化协调同步，扎实开展项目建设攻坚年、招商引资推进年、全民创业服务年、基础设施提升年活动，切实保障和改善民生，全面提升公共服务水平，奋力开创科学发展、跨越式发展新局面。

　　2013年主要预期目标是：生产总值增长13%；全社会固定资产投资增长31%；社会消费品零售总额增长18%；地方公共财政预算收入增长16%；城镇居民人均可支配收入、农民人均纯收入分别增长13%；城镇登记失业率控制在4.5%以内；人口自然增长率6.5‰以内；居民消费价格涨幅4%左右；万元生产总值能耗、二氧化碳和主要污染物排放等控制性指标达到省定要求。

　　围绕上述总体要求和预期目标，重点做好以下工作：

　　（一）围绕建设经济强市，全面提升产业实力。

　　以“三大工程”〔14〕为抓手，推动工业快速发展。把扩大工业经济总量作为主攻方向，把做大做实做强工业落实到产业、园区、企业和项目上，形成大项目“顶天立地”、小企业“铺天盖地”的良好局面，发展壮大实体经济。力争规模以上工业增加值增长21%。做大做强骨干支柱企业，做实重点产业集群。支持三江集团民品加速发展，支持华中光电汽车零部件基地建设，支持永祥粮机、康欣木业等企业上市。力争新增销售收入过10亿元企业5家、过30亿元企业2家，新增百亿产业集群1个。推动中小企业快速成长，选择一批成长性好的中小企业进行重点培育，加强品牌建设、市场开拓，力争新增规模以上企业100家以上。加大小微企业孵化力度，力争孵化企业1000家以上。大力培育和发展光电子信息、新能源、新材料、生物医药、节能环保等战略性新兴产业。

　　以深化农村综合改革为核心，推动农业加快发展。认真落实强农惠农富农政策，有效整合项目资金，扎实开展新农村“一线一点”和新一轮“三万”活动。创新经营体制机制，着力培育一批专业大户、家庭农场、专业合作社等新型农业经营主体。大力推广“春晖模式”〔15〕，加快土地流转步伐。发展壮大特色板块基地。继续实施农产品加工业“四个一批”〔16〕工程，扶持孝棉集团、银鹭食品、神丹食品、益嘉林业、鸿翔肉鸭、悟道茶等重点农业产业化龙头企业发展壮大，加快孝南现代森工、安陆宝迪食品等十大农业产业化项目建设。建立市农产品质量安全监测中心，确保让人民群众吃上放心粮、放心肉、放心菜。

　　以现代服务业为重点，推动第三产业突破性发展。创新发展物流、金融、保险、中介、信息等生产性服务业。高质量建设现有物流园区，引进一批重点物流企业，打造物流节点城市。鼓励引进金融机构，推进农村金融全覆盖。大力发展商贸、旅游、文化、家政等生活性服务业。加快完善覆盖全域的商贸服务体系，发展壮大一批商贸企业。引进国内20强地产企业进行综合开发。着力提升5个国家4A级景区的品位和档次，加快凤凰七仙城等旅游重点项目建设。大力发展电子商务、网络购物、服务外包、托幼养老、租赁消费等新型消费业态，培育新的消费增长点。

　　以信息化融合为路径，推动“四化”同步发展。加快推进信息化和工业化深度融合，以信息化促进城镇化和农业现代化。抓好工业化、信息化“两化”融合试点，加强农村信息基础设施建设，加快培育信息服务企业，建设孝感软件产业园。加强信息技术在政务商务、生产生活、社会管理等领域的深度应用，加快推进“三网融合”〔17〕，建设数字城市〔18〕、数字企业〔19〕。

　　（二）围绕建设鄂豫省际区域性中心城市，全面提升新型城镇化质量。

　　高起点规划。充分利用孝感交通、区位、资源、人才等优势，与周边省际城市竞相发展，助推武汉城市圈副中心城市建设。进一步完善孝汉一体、市区一体、云孝一体、城乡一体全域孝感规划体系，全面打通四级规划，提升各类规划。完成孝感中心城区总体规划修编及相应专项规划，完成各县市城区总体规划修编，完成重点镇和中心村（社区）控制性详规，完成“一线一点”控制性规划。切实维护规划的严肃性，严格按规划建设，加强规划全过程管理。

　　高标准建设。坚持产城融合、双轮驱动，有序推进中心城市、县级中等城市、重点镇、中心村（社区）建设。围绕“六大组团”，加快孝感中心城市建设。全面推进东城新区行政、文化、商住、金融等项目和市政工程建设，加快行政事业单位搬迁和片区开发步伐，掀起东城新区建设高潮。支持县市老城区改造和新区建设。以产业发展和人居环境改善为重点，抓好30个重点镇和100个示范中心村（社区）建设。加快基础设施“六张网”〔20〕和社会事业“六张网”〔21〕的延伸覆盖，新建城市、城镇、农村三类新社区100个以上。

　　高水平管理。积极创建全国文明城市、国家环保模范城市、国家卫生城市、国家园林城市和文明卫生村镇，广泛开展全民参与、共建共享、文明教育、示范创建四大行动。推进城管体制改革，构建“大城管”格局，建立长效机制。加快社区网格化管理、小区物业管理“两个全覆盖”向县市城区和集镇延伸。有序推进农业转移人口市民化。

　　(三)围绕建设中华孝文化名城，全面提升文化软实力。

　　着力打造孝文化品牌。加强孝文化载体建设，以槐荫公园、澴川公园、黄香文化园、孟宗公园为重点，建设一批孝文化标志性工程。充分挖掘地方特色文化，壮大“一县一品”文化品牌。加强孝德、孝廉教育。举办第十届孝文化节，提高中华孝文化名城知名度和影响力。

　　发展壮大文化产业。编制文化旅游产业发展规划，策划一批高质量的文化产业项目。不断完善文化体制改革，组建广电传媒集团、报业传媒集团，支持现有文化龙头企业做大做强。促进文化与科技、旅游、养老产业融合，建设孝文化旅游圈带，加快“中华敬老园”、“全国爱心护理院”建设。

　　不断丰富人民群众文化生活。加强县市区“三馆”和社区文化设施建设，加快建设市文化中心。实施“文化惠民工程”，新增一批农民体育健身器材。组织开展建市20周年系列文化活动，举办民间文艺大赛和第八届楚剧展演。积极发展网络文化，鼓励文艺创作，加强传统文化的保护与传承。着力推进社会公德、职业道德、家庭美德、个人品德建设，进一步提高市民素质。

　　（四）围绕建设创新城市，全面提升发展活力。

　　全力推进两型社会建设。坚持先行先试，在资源节约、环境保护、城乡统筹等领域的改革创新上不断取得新突破。支持县市两型社会示范区建设。以国家高新区、临空经济区为载体，加强与武汉战略合作，加大融入力度，加快建设孝汉城际铁路、白水湖大桥、硚孝高速等重点对接项目，在基础设施建设、产业转移、区域市场、城乡建设、环境保护“五个一体化”上取得新进展。

　　全力推进大别山试验区建设。加快大悟、孝昌试验区建设步伐，全面启动安陆、双峰山试验区建设。支持整合使用政策性资金，统筹推进试验区现代农业示范、城乡统筹、生态家园等“点中点”项目建设。积极配合全省鄂北地区水资源配置工程〔22〕建设。南部县市区对口帮建大悟、孝昌1个乡镇，市直新农村建设成员单位对口帮建试验区1个村。

　　全力破解瓶颈制约难题。创新融资办法，做大融资平台，拓宽融资渠道，构建多元化融资格局。坚持集约节约用地，用足用活用好国家土地政策，加强对闲置低效利用土地的清理处置。实施槐荫人才计划〔23〕，坚持培养使用和招才引智结合，着力培养、引进一批行业领军人才和实用人才，造就一批追求卓越、敢闯善干、有强烈社会责任感的企业家队伍。以建设城镇新社区为突破口，破解拆迁安置难题。

　　全力推进市直“三区”跨越式发展。探索建立与市直“三区”目标定位相适应的管理体制。做实做大做强孝感高新区，发挥国家高新区品牌效应，力争引进世界500强、国内500强企业3—5家、央企或行业龙头企业10家，新签约、开工、竣工项目各30个。全面推进临空经济区基础设施“十大工程”〔24〕，加快建设现代物流园，启动建设高新技术产业园、文化创意产业园，力争合同引资额突破150亿元。提升双峰山旅游度假区整体形象，积极争创国家5A级景区，力争接待游客人数突破50万人次。

　　全力支持县域经济加快发展。全面落实推进各县市区科学发展、跨越式发展座谈会和三级干部会议精神，强化政策激励，帮助解决困难问题，不断实现赶超进位。支持孝南发挥市区一体优势，奋力冲刺全省县域经济二十强；支持汉川奋力冲刺全省县域经济十强；支持应城建设全省经济强市、全国文明城市；支持云梦加快云孝一体化、城乡一体化步伐，建设全省经济强县；支持安陆努力走在全省县域经济第二梯队前列，奋力冲刺全省县域经济三十强；支持大悟全力打造高铁新城，争当大别山经济社会发展的“排头兵”；支持孝昌建成全省山区经济强县。

　　（五）围绕建设人民幸福城市，全面提升人民群众生活水平。

　　促进就业更充分。坚持就业优先，促进创业带动就业，积极创建国家级创业型城市。全面落实各项扩大就业政策措施，实现就业培训、就业服务和就业援助三大体系全覆盖。培育市场主体和创业平台，拓宽就业渠道。积极帮助解决高校毕业生、农村转移劳动力等特殊和困难群体就业问题。力争新增城镇就业4.3万人，下岗失业人员再就业1.5万人，农村劳动力转移就业3.9万人。

促进保障更全面。构建覆盖城乡的社会保障体系，完善医疗、生育、工伤保险市级统筹。健全城乡低保动态管理机制。扎实做好灾害应急救援和生活救助。建立健全弱势群体长效救助机制，加快推进残疾人保障和服务体系建设。实现县市区社会福利中心全覆盖。加大保障性住房建设、分配和管理力度，新增保障性住房5600套，廉租住房租赁补贴2700户，改造各类棚户区1.06万户，农村危房5000户。继续扩大住房公积金制度覆盖面。有效落实调控政策，推动房地产业平稳健康发展。

　　促进社会事业更繁荣。鼓励科技创新，争创国家科技进步先进城市“五连冠”。办好学前教育，支持特殊教育，推进义务教育均衡发展，推动普通高中教育优质化、特色化。加快发展现代职业教育，推动湖北工程学院、湖北职院内涵式发展。深入推进公立医院改革，巩固基本药物制度，放开引进优质医疗资源，提高基本公共卫生服务均等化水平。加强人口计生信息化建设，下大力控制出生人口性别比。扎实做好第三次全国经济普查工作。深入开展双拥共建，积极支持在孝部队建设。加强国防教育、人民防空和民兵预备役建设。全面落实民族宗教政策，做好外事侨务、对台、老龄、地方志、档案、保密等工作，支持中央、省驻孝单位建设和发展。

　　促进社会管理更到位。建立和完善社区综合服务、综治信访维稳、网格化管理三个中心，加强社区工作者、网格管理员、社区志愿者三支队伍建设，完善三个“1+X”服务管理体系。健全领导干部接访下访长效机制，完善人民调解、行政调解、司法调解联动工作格局。全面实施警务机制改革，集中整治突出治安问题。建立健全突发事件应急联动机制。强化企业主体和部门安全监管责任，加大隐患排查治理力度。加强价格监管，构建食品药品和产品质量安全追溯系统，让人民群众用放心药物、吃放心食品、过放心生活。

　　促进生态环境更优美。把生态文明建设放在突出位置，努力建设美丽孝感。严格实施市域空间主体功能区规划，对王母湖、野猪湖、白水湖、汈汊湖实行保护性开发。加快建设槐荫公园三期、邓家河生态湿地公园一期、城西森林公园、仙女湖公园等一批生态项目，孝感城区每个街道建设一处1500平方米的绿地。严格落实节能减排责任制，加快实施重点行业、重点企业节能减排项目，深入推进云应地区盐化工、汉川火力发电、黄麦岭磷化工三大循环经济园区试点工程。大力推进绿色建筑，积极申报全国绿色生态城区。加强耕地、水资源、环境保护，综合整治农村生态环境。

　　（六）坚持突破重点、夯实基础，为加快建设“五个城市”提供实力支撑。

　　扎实开展项目建设攻坚年活动，强化项目支撑。完善“六个一”〔25〕的推进机制，在项目策划、争取、落地、建设、竣工、投产达产上实施攻坚。深入研究国家投资政策，主动与国家部委和省直单位对接，争取更多支持。加强与知名企业、院校、科研院所的联系，寻求广泛合作。力争策划申报项目700个以上，争取国家、省资金高于上年水平。加大项目建设督查力度，强力推进100个事关全局的重大项目建设。

　　扎实开展招商引资推进年活动，强化产业支撑。坚持投资强度、税收额度、科技高度、链条长度、环保程度“五度”原则，全面主动研究招商对象的产品、市场、潜力，实行招商项目第三方评估，确保引进真项目、好项目。对已签约项目明确责任主体，实行全程跟踪服务，提高履约率和开工率。紧盯央企、上市公司、行业龙头企业，力争中国交通建设集团投资100亿元基础设施项目尽早开工建设，争取中国广东核电集团、广东物资集团、恒大集团落户孝感，加强与中国邮政集团、中国电子信息产业集团、港中旅集团等大型企业集团跟踪洽谈。加强与国内外友好城市交往合作，推动对外开放向深度和广度迈进。扎实开展全民创业服务年活动，强化环境支撑。制定出台鼓励全民创业、促进民营经济跨越式发展的政策措施，大力营造创业兴业环境，加快民营经济发展步伐。新培育各类市场主体2.5万户，民营企业2000家。

　　扎实开展基础设施提升年活动，强化基础支撑。不断提升工业园区承载力。抓好园区基础设施建设，新建各类园区道路212公里，配套管网190公里，拓展园区面积116平方公里。不断提升农业基础设施。实施水利建设七大工程〔26〕，新增有效灌溉面积30万亩，治理水土流失面积70平方公里。不断提升城市功能。投资71亿元，加快孝感城区112个重点城建项目建设步伐。加快北京南路建设进度，完成文化西路、长征北路等改造工程，实施乾坤大道、黄陂东路、天仙北路延伸工程，开工建设城际铁路东站广场。不断提升交通条件。加快316国道和107国道改扩建、武汉城市圈环线高速孝感段、麻竹高速大悟段、孝汉应快速通道等重大交通项目建设。

　　各位代表，市政府将继续办好改善民生“十件实事”。（1）新改扩建幼儿园30所，实施孝感师范和楚澴中学改造工程。（2）启动市中心医院全科医生临床培养基地建设，新改扩建7个县级医疗机构和17个乡镇卫生院。（3）新建万人千吨集中饮水工程12处，铺设农村供水管网1200公里，新解决农村25万人饮水安全问题；完成农村清洁新能源入户4万户。（4）新建30公里一级公路、80公里县乡公路、400公里通村公路；推进城区公交、出租车经营管理体制改革；孝感城区公交系统使用敬老IC卡。（5）建设八一水厂及临空经济区供水主干管网，完成人民广场二期、槐荫公园二期、污水处理厂二期工程。（6）在孝感城区新建专业停车场2处，新建视频监控探头1000个以上；高标准改造金西门市场、严桥市场。（7）加快建设市福利康复中心，新改扩建20所农村福利院。（8）建设市食品药品电子监管系统和公共检测平台。（9）免费实施1000例白内障患者复明手术，免费发放残疾人辅助器具3000套。（10）免费办理法律援助案件1000件以上，办理其他法律援助事项1万件以上。

　　三、切实加强政府自身建设

　　新的形势、新的目标，对政府工作提出了更高要求。我们将始终坚持服务为本，提能善政，努力建设人民满意的服务型政府，以更高更强的精神区位、只争朝夕的进取精神、不胜不休的决战气魄，为加快建设“五个城市”提供坚强有力的保障。

　　（一）深入解放思想。认真贯彻落实党的十八大、省十次党代会和市委五届四次全体（扩大）会议精神，深化“解放思想大讨论”、“纠风正纪促跨越”活动，按照“竞进提质”的总要求，牢牢把握“绿色GDP”和“民生GDP”两个要点，在政府系统全面形成敢于突破条条框框、敢于让利求发展的担当精神，形成敢与高的比水平、敢与快的比速度、敢与强的比干劲的拼搏精神，形成敢于动真碰硬、勇于攻坚克难、善于突破瓶颈的创新精神。市政府领导带头解放思想，一级带着一级干，一级做给一级看，营造科学发展、跨越式发展的浓厚氛围。

　　（二）坚持依法行政。牢固树立法治意识，加强法治政府建设，运用法治思维和法治方式深化改革、推动发展、化解矛盾、维护稳定。自觉接受市人大的法律监督、工作监督和市政协的民主监督，认真执行市人大及其常委会的决议决定，积极支持政协委员发挥参政议政作用，高质量办理人大代表建议和政协委员提案。加强行政监察和财政、审计监督，强化新闻舆论、社会公众监督，广泛听取各民主党派、工商联和无党派人士的意见建议，积极支持各人民团体开展工作。建立健全决策程序，推进科学决策、依法决策、民主决策、高效决策。

　　（三）高效抓好落实。空谈误事、实干兴业。认真落实中央关于改进工作作风、密切联系群众的“八项规定”〔27〕，在政府系统形成雷厉风行、多做少说、务实高效的作风，自觉做到勤于谋事心到位、深入实际身到位、全力以赴力到位。落实“三短一简”，坚持勤俭节约。倡导“一线工作法”，重心下移，把精力放在抓招商、跑项目、下基层、解难题上。完善政务服务体系，推进行政审批“三集中三到位”〔28〕，启动市本级政务服务大楼建设。增强担当意识，做到小事不拖拉、难事不回避、好事不揽功、大事集体决策，定下的事强力推进，看准的事一抓到底，群众反映的事马上落实。

　　（四）严格廉洁从政。全面推进惩治和预防腐败体系建设，做到干部清正、政府清廉、政治清明。推进民主评议政风行风常态化。加强政府重大项目建设、重大财政支出、各项惠民资金的全程监管和审计监督。稳步推进公务卡制度改革，建成覆盖市县两级的电子监察综合管理平台，建立统一规范的公共资源交易市场。健全对权力运行的制约和监督机制，严惩公权滥用，加大腐败案件查处力度，以反腐倡廉的实际成效取信于民。

　　各位代表，成绩令人鼓舞，目标催人奋进。让我们在市委的正确领导下，以更加坚定的信心、更加昂扬的斗志、更加务实的作风，激情奋进，克难攻坚，加快建设武汉城市圈副中心城市，打造“湖北的苏州”，建设“魅力孝感”，为全市人民过上更加幸福美好的生活而努力奋斗！
